# Historical and Clinical Experiences of Gene Therapy for Solid Cancers in China

**DOI:** 10.3390/genes8030085

**Published:** 2017-02-24

**Authors:** Bo Li, Ning Gao, Zhuang Zhang, Qian-Ming Chen, Long-Jiang Li, Yi Li

**Affiliations:** 1 State Key Laboratory of Oral Diseases, National Clinical Research Center for Oral Diseases, West China Hospital of Stomatology, Sichuan University, Chengdu 610041, China; li_yi@scu.edu.cn (B.L.); gaoningdaniell@163.com (N.G.); zhangzhuang1949@163.com (Z.Z.); qmchen@scu.edu.cn (Q.-M.C.); 2 Department of Head and Neck Oncology, West China Hospital of Stomatology, Sichuan University, Chengdu 610041, China

**Keywords:** gene therapy, history, clinical trial

## Abstract

Based on the theoretical and clinical development of modern medicines, gene therapy has been a promising treatment strategy for cancer and other diseases. The practice of gene therapy is nearly 27 years old, since the first authorized gene transfer study took place at the National Institute of Health in 1989. However, gene therapy was not readily adopted worldwide, until recently. Several gene therapy clinical trials have been carried out in China since 1998, and medical research in China has flourished. In this report, we review the history of gene therapy in China, focusing on treatment protocol, the administration cycle, dosage calculation, and the evaluation of therapeutic effects, in order to provide more information for the additional development of this promising treatment strategy.

## 1. Global and Chinese History of Gene Therapy

### 1.1. Brief History of Gene Therapy in the World

Since deoxyribonucleic acid (DNA) was identified as hereditary material, gene transfer has become a focus of medical researchers. Gene therapy was developed, based on the theories of genetic inheritance. Gene therapy is a therapeutic strategy that aims to replace faulty genes with normal, functional genes in the patients’ genome, in order to treat diseases caused by genetic defects with drugs administered by viral vectors or genetically engineered microorganisms [1]. In 1966, Edward Tatum discussed the possibility of gene therapy by using viruses as the vectors in somatic cells [2]. Based on the theories of Tatum, an initial proof-of-concept of virus-mediated gene transfer was demonstrated by Rogers et al., who implemented the first gene therapy trial. In that trial, the authors used the wild-type (WT) Shope papilloma virus to transfer the arginase gene into two female pediatric subjects suffering from a urea cycle disorder, but the results were negative [3]. In 1972, an article entitled “Gene therapy for human genetic disease?” was published in Science by Theodore Friedmann and Richard Roblin, which cited Rogers’ proposal of 1970, that “good DNA” could be used to replace defective DNA in people with genetic disorders [4]. However, there were no major developments in gene therapy until 1989, when the first clinical trial was carried out in two children at the NIH Clinical Center, by W. French Anderson from the National Heart, Lung and Blood Institute [5]. In that pivotal study, the patients suffered from a congenital disease known as adenosine deaminase (ADA) deficiency, which severely affects immunity and the ability to fight infections. As part of the treatment, Anderson obtained white blood cells (WBCs) from the patients, replaced the defective gene with the ADA gene, and then re-administrated the WBCs to the patients. Even though one patient responded to the gene therapy, there was some debate about the results, because an enzyme replacement therapy was synchronously given to the patient with polyethylene glycol ADA [5]. An adenosine deaminase-severe combined immune deficiencies (ADA-SCID) trial was conducted in the EU by Bordignon C. et al., and those results indicated successful gene transfer in long-lasting progenitor cells, the immune repertoire of the patients was normalized, and their humoral immunities were restored after two years of treatment [6]. Gene therapy trials have also been conducted for other diseases, such as hemophilia, various severe combined immune deficiencies (SCIDs), and cancer. Mukhopadhyay and Roth initially showed the feasibility of p53 gene therapy in 1993, by transferring the WT p53 gene into the H322a cell line (human non-small cell lung cancer (NSCLC)), in order to examine the role of the WT and mutated p53 genes during cell growth and tumorigenesis [7]. The first p53 recombinant adenovirus was reported by Zhang et al., who introduced WT p53 by Ad5CMV-p53 into four human NSCLC cell lines, in order to show the promising tumor-inhibiting effects of p53 gene therapy [8]. Based on these preclinical studies, the first clinical trial of p53 gene therapy was carried out by Roth et al., who directly administrated a retroviral vector containing the WT p53 gene into nine NSCLC patients for whom conventional treatments had failed, and the results showed tumor regression in three of the patients and a stabilization of tumor growth in a further three patients [9]. Gene therapy was also employed to fight genetic disease. The first clinical trial was initiated in 1999, on X-linked SCID (SCID-X1), and the results were promising: Gamma-c transgene-expressing T and NK cells were detected in two patients, and the counts and function of T, B, and NK cells were improved in the patients who received experimental treatment, when compared to those in the control group [10]. Unfortunately, the trials were halted in 2003 due to the development of T cell acute lymphoblastic leukemia (ALL), caused by the retrovirus vectors. However, gene therapy trials have not been devoid of fatal outcomes. In 1999, the first patient died as a result of the viral vector used in the gene therapeutic reagents. The 18 year old male patient took part in a gene therapy clinical trial for deficiency of ornithine transcarbamylase at the University of Pennsylvania in Philadelphia. A severe side effect, that of multi-organ failure, resulted in the subject’s death four days later, caused by an immune reaction immediately after a very high dose of adenovirus administration [11]. After this tragic event, gene therapy clinical trials were halted in both the USA and EU.

On 19 July 2012, a gene therapy drug known as Glybera (Amsterdam Molecular Therapeutics, later marketed by UniQure), was approved by the European Medicines Agency. Glybera is an adeno-associated viral (AAV) vector, used to treat severe lipoprotein lipase deficiency by expressing the lipoprotein lipase in the muscle tissue. The approval of Glybera marked a new era of global gene therapy development, especially relating to the aspects of the regulatory perspective. Several challenges in the regulation of gene therapy were discussed in a recent editorial [12], which were demonstrated to be resolved by the approval of Glybera. Improved viral vectors have been developed to promote gene therapy. A self-inactivating γ-retrovirus vector was utilized in a clinical trial to transduce autologous CD34+ hematopoietic stem cells with the IL-2Rγ gene for transplantation in SCID-X1 patients, and although a long-term follow-up period is needed, the treatment showed some early clinical benefits [13]. Strimvelis (GlaxoSmithKline, London, UK) was approved by the European Medicines Agency (EMA) on 27 May 2016, based on clinical trials which showed a 100% survival rate, and a median follow-up time of seven years for ADA-SCID patients [14]. In October 2015, Talimogene laherparepvec (T-Vec, trade-named Imlygic, and formerly known as OncoVexGM-CSF) was approved bt the US FDA to treat melanoma, before also being approved by the EMA in December 2015; an event that marked the first approval of an oncolytic virus in the West. T-Vec is a biopharmaceutical drug which is injected directly into melanoma lesions that cannot be surgically removed. As of 2016, there has been no evidence that T-Vec extends the life of people with melanoma, or that it prevents metastasis [15]. However, there have been huge successes in clinical trials with genetically-modified T cells expressing chimeric antigen receptors (CAR-T) [16]. In CAR-T therapy, T cells are collected from the patient’s own blood, and genetically engineered to produce special receptors (CARs) on their surface, allowing the T cells to recognize a specific antigen on tumor cells. After expansion in the laboratory, CAR-T cells are then infused in the patient to proliferate, and with guidance from their engineered receptor, they can recognize and kill cancer cells that harbor the specific antigen on their surfaces. CAR-T efficacy has been demonstrated in a range of hematological cancers and multiple myeloma [17], and by some encouraging clinical data reported in early phase I trials in solid tumors, including neuroblastoma and tumors overexpressing mesothelin, HER2, and EGFR [18,19,20,21].

**Figure 1:**
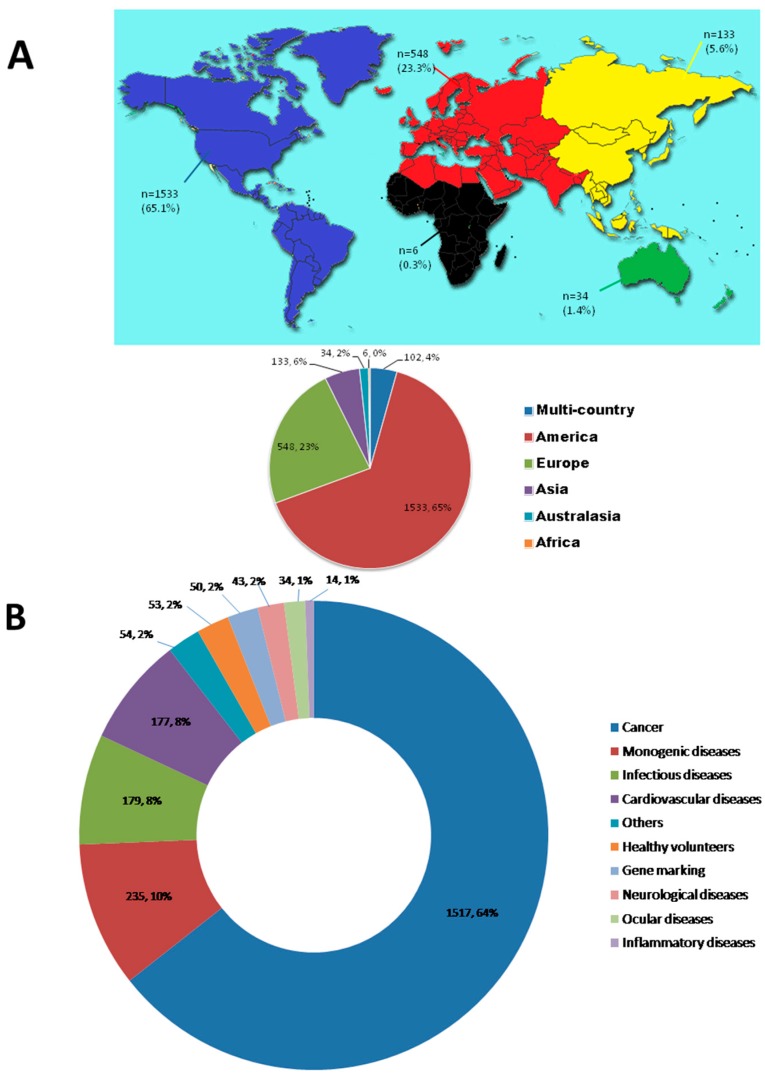
Distribution of Gene Therapy Clinical Trials. (**A**) Geographic distribution of gene therapy clinical trials by continent; (**B**) distribution of gene therapy clinical trials by indication. Data cited from The Journal of Gene Medicine, www.wiley.co.uk/genmed/clinical.

Until 2016, 2356 approved gene therapy clinical trials had been conducted worldwide, or are still ongoing (Figure 1). Adenovirus, retrovirus, and naked plasmids have been the most common gene transfer vectors in clinical trials. A total of 1517 clinical trials have been conducted, aimed at treating various cancers, marking cancer as the most common condition currently targeted with gene therapy. Furthermore, the trials that have been conducted or are still ongoing in the continents of America and Europe, have been as a result of the approval of the FDA and/or EMA to restart gene therapy (data cited from The Journal of Gene Medicine, www.wiley.co.uk/genmed/clinical). Current data indicate that we are in a new era of gene therapy development.

### 1.2. Brief History of Gene Therapy in China

Although gene therapy clinical trials were blocked before 2012 in the United States and the European Union, they developed pretty successfully in China, due to the support of the government and the Chinese Food and Drug Administration (SFDA). A stage I clinical trial of recombinant adenoviral p53 (SBN-1) was carried out from 1998 to 2003, in four hospitals in Beijing, and is believed to be the beginning of Chinese gene therapy [22]. In 2003, the first gene therapeutic reagent was approved by the SFDA, and China became the first country to approve a gene therapy-based product for clinical applications. The recombinant adenoviral p53 (rAd-p53, Gendicine™, ShenZhen SiBiono Gene Tech, Shenzhen, China) is a recombinant human serotype 5 adenovirus, in which the E1 regions have been replaced by a human WT p53 expression cassette. As a consequence, rAd-p53 limits the infectivity of the virus to only one cycle, and the adenovirus genome does not integrate itself into the host genome DNA, nor impair the functionality of normal cells [23]. Between 2000 and 2012, all gene therapy clinical trials were carried out in China, and Chinese researchers and physicians acquired a lot of experience in the associated procedures. A total of 16 controlled clinical trials of rAd-p53 (Gendicine^TM^) have been carried out for the treatments of advanced cancers, including head and neck cancer, hepatic cell carcinoma, NSCLC, malignant glioma, and epithelial ovarian carcinoma [22,24,25,26,27,28,29,30,31,32,33,34,35,36,37,38] (Table 1). Overall, the outcomes of the present clinical trials have been satisfying, and the overall response rates and survival rates were better in the rAd-p53 treatment groups, than those in the control groups. For example, Zhang SW’s group reported the results of a combined treatment of gene therapy and radiotherapy for the patients of nasopharyngeal cancer in 2009: the local control rate in the combined treatment group (39.7%) was 10-fold higher than the radiotherapy-only group, the five-year survival rate of the combined group was 13.1% higher than the control group (*p* < 0.01), and the disease-free survival rate was 29.7% higher (*p* < 0.01) [31]. Guan used rAd-p53, combined with chemotherapeutic drugs, to treat advanced NSCLC via bronchial arterial access in a controlled clinical trial, and the results showed that the combined group witnessed a longer time without progression (*p* = 0.018), but survival analysis showed no statistical difference (*p* = 0.224) [25]. Guan also used rAd-p53 in hepatic arterial chemoembolization (TACE), and the total effective rate was 58.3% for the combined group, and 26.5% for the TACE-only group (*p* < 0.05). The patients in the combined group had a lower incidence of gastrointestinal symptoms (*p* < 0.05), and a higher survival rate (*p* = 0.0002) [26]. Li et al. administrated rAd-p53 and chemotherapeutic drugs to the patients of advanced oral squamous cell carcinoma, via selective intra-arterial infusion in a randomized, controlled clinical trial, and the patients in the combined group had a significantly higher survival rate than either the chemotherapy-only or gene therapy-only group (*p* = 0.019), and the most frequent vector-related complication was a transient fever [27].

Compared to Gendicine™, phase 1 and 2 clinical trials were carried out to evaluate Ad5CMV-p53 (INGN 201; ADVEXIN, Austin, TX, USA), a similar adenoviral p53 gene therapy reagent developed by Introgen in the US, for use in local advanced bladder, lung, head and neck cancer, and glioma by intra-tumoral injection [39,40,41,42,43,44]. All of the results showed that the intra-tumoral injection of ADVEXIN could transfer the exogenous p53 gene to the cells, and that positive clinical responses were observed in the treatment groups, including a minimal toxicity. Thus, the gene therapy with ADVEXIN was effective and safe, similar to the results of Gendicine™. Two phase III clinical trials were consequently developed, based on the results of the phase 1 and 2 clinical trials, and one was completed. In that completed study, the researchers found that patients with either WT p53 or a low-level expression of mutated p53, were associated with a significant increase in survival, when compared to patients with an unfavorable p53 profile (7.2 months vs. 2.7 months, *p* < 0.0001) [45]. Based on the results of ad-p53 clinical trials, we can conclude that the gene therapy is a safe and promising treatment strategy for advanced cancer patients, depending on optimal treatment design and drug administration.

## 2. Clinical Issues and Experiences of Gene Therapy

### 2.1. The Combined Therapy and the Sequence of the Treatment Protocol

Chemo- and radio-therapy, recognized as traditional and classic forms of cancer treatment, ionize DNA or generate free radicals to induce DNA lesions, including single- and double-stranded breaks and base damage [46]. However, clinical results have shown that these two treatment strategies do not yield satisfactory results for patients, especially for solid tumors, which may explain the lack of significant improvements in the overall survival rate over the last 30 years [47]. With the development of gene therapy, we have found that this alternative form of therapy can play an important role in the combined therapeutic strategy [27]. For example, a synergistic relationship between p53, and chemo- and radio-therapy has been suggested. Cells have a precise regulating system for radiation or chemo-induced DNA damages, and the p53 gene is known to play an essential role in the DNA repair system [48]. The profiles of gene activation, depending on the p53 gene, can drive tumor cells toward apoptosis or cell cycle arrest from a molecular perspective, and the profiles are post-translationally modified by p53, activated by DNA damage [49]. Based on these mechanisms, tumor cells can be maximally killed, while normal tissues are preserved. Thus, the p53 gene may be useful as a target for pharmacological inhibition to reduce side effects, especially in cancer patients with mutant p53 [49,50].

The status of p53 and its gene family could regulate the chemo-sensitivity of cancer cells via senescence and bystander effects, which is defined as the killing or damaging of cells that have not directly received chemotherapy or irradiation, through the diffusion of soluble death-promoting factors from targeted cells. Like radiotherapy, the main pharmacological effects of the cytotoxic chemotherapeutic agents are aimed at DNA damage [49,51]. In chemo- and radiotherapy, similar signaling cascades are involved in the p53 activation of DNA damage, and the WT p53 gene plays an essential role in inducing apoptosis. It has been demonstrated that there were relevancies between the mutant p53 genes and resistance to chemotherapy in ovarian cancer [52], gastric and colorectal cancers [53], and in hematological malignancies [54]. In addition, dramatic differences were shown, indicating that the p53 status in tumors was not related to the chemotherapy response, but rather, that mutations in L2/L3 domains of the p53 gene in breast cancer were related to chemo-resistance [55]. Sakai reported that a significantly increased expression of TP53 was observed in the post-treatment samples following chemoradiotherapy. These results suggested that chemotherapy and/or radiation exert a physiological selective pressure on tumors, which leads to the acquisition of TP53 mutations [56]. Therefore, it is meaningful in clinical practice to combine chemo- and radiotherapy with rAd-p53 gene therapy in order to correct the gene defects and mutations, which should reduce the treatment resistance and produce other benefits for cancer patients. Furthermore, the WT p53 protein could mitigate the toxicity of chemotherapeutic drugs by at least one of the following pathways: (1) p53 proteins interacting with DNA helicase; (2) increasing of ribonucleotide reductase by p53; and/or (3) the 3′→5′ exonuclease activity of the p53 protein [27].

The basic theories of radio- and chemotherapy are significantly different from those of gene therapy, and all of these treatment strategies could complement each other [57,58]. In clinical practice, gene therapy drugs could be administered twice to concentrate the cancer cells in the G1 cell cycle stage, and this could then be followed by chemotherapy and/or radiotherapy in order to achieve better results. At the same time, it is meaningful to reconstruct the p53 pathway by gene therapy before treatment, which is called a “cellular preparation”, which could minimize therapeutic resistance [27,59].

### 2.2. Dosage Calculation and Routes of Administration in Gene Therapy

The multiplicity of infection (MOI) refers to the ratio of agents, such as viruses or bacteria, to the targeted cells. A successful transduction of the gene therapy reagents would be approved by a sufficient MOI. The virus particles should be sufficient enough to surround the target cells to ensure effective infection ratios. An optimal MOI is essential in the dosage calculation for the administration of gene therapeutic drugs, since high viral particle doses can induce severe hepatic damage, even resulting in the death of patients [60]. Traditional dose determination methods, such as those based on the body surface area or the dosage per kilogram of body weight, as used in chemotherapy, may not be ideal for gene therapy. Until now, there is no clinically accepted method for calculating the dose in gene therapy due to the following reasons: (1) the most accurate method should be dependent on the total number of cancer cells, which are calculated by the tumor volume and the average cell density in the primary focus, both of which cannot be decided according to the tumor invasion and the heterogeneity of the primary focus; and (2) it is also important to decide on the minimal number of vectors required for successful transfer, since cells may have different sensitivities to viral particles [61]. Therefore, while accurate dose calculation cannot be currently achieved, researchers can use estimated cell numbers, combined with the best titer for each cell type, to calculate the dose for gene therapy. The dosages in the previous clinical trials of rAd-p53 varied dramatically, from 1–4 × 1012 viral particles per site [8,22,24,25,26,27,28,29,30,31,32,33,34,35,36,37], which were calculated based on the tumor sizes, the medical direction, and the researchers’ personal experiences. Li et al. reported that when the MOI was 100, rAd-p53 infection could effectively introduce an exogenous p53 gene into POE-9n cells, and decrease the toxicity and side effects of the adenovirus [62]. Based on the differences in the cells of the solid cancer from various organs, preclinical research on the effective infection titer for gene therapy should be conducted before the clinical trials.

The doses used in gene therapy should also be different, according to the routes of administration [27,59]. An intravenous infusion is only suitable for patients with widespread metastasis, but the dosage for the treatment should be increased, which can paradoxically increase the possibility of relevant side effects; at the same time, the transfection efficiency of gene therapy could be questioned. Due to the neutralizing antibody-mediated attenuation and the first pass-elimination by the liver, rAd-p53 could be diminished before reaching the tumor focus, when vectors are administrated via intravenous infusion. Meanwhile, with the development of immunotherapy, the treatment effect via intravenous infusion is likely, due to the adenovirus-mediated delivery of the WT p53-induced p53-specific immune response, via DCs (Dendritic cells) and sensitized SCLC (Small Cell Lung Cancer), subsequent to chemotherapy. However, an examination of this hypothesis is needed to clarify the anti-tumor mechanism in this procedure. Due to the hepatotoxicity of the viral vectors, a high dose to ensure MOI should be avoided in the clinical trial. It may be a good idea to increase the therapeutic dose of gene therapy by local administration, including intra-tumoral injection, intra-arterial infusion, and perfusion [8,22,24,25,26,27,28,29,30,31,32,33,34,35,36,37,38]. While intra-tumoral injections and intravenous infusion are simple procedures, there were some shortcomings when using these administration routes [23,63]: (1) it is not easy to cover the whole tumor of the anatomical position via intra-tumoral injection, which might result in treatment blind spots; (2) the drugs may not sufficiently reach the hard tissues where the tumor invades; and (3) transmission of the vectors from the injection site over the tumor focus areas may not be easily achieved [61,64]. Compared to previous studies, in which the intra-tumoral or intravenous infusion of rAd-p53 was applied, there were better overall clinical responses in Li’s study [27], which applied intra-arterial administration. The results of the Kaplan-Meier plot from that study showed that the patients in the rAd-p53-only group had a longer median survival time than those in the study by Clayman et al. [26,65]. The following factors may have resulted in the better clinical outcomes of Li’s trial. Firstly, the drugs of gene therapy and/or chemotherapy were applied through selective arteries in a retrograde manner, which could target the primary focus area more accurately and increase the local dosage. At the same time, it could also help deliver the drugs sufficiently, throughout the tumor. Secondly, there are definitely more anatomical blood feedings in the head and neck regions, which makes the delivery of drugs more accurate to the target tumors. Thirdly, retro-arterial administration could reduce the side effects by decreasing the effects on the non-relevant organs, by decreasing the dose of the therapeutic reagents. Finally, the drugs will initially reach the primary focus and adjacent tissues by intra-arterial administration, and will not be diminished by the liver, so a comparatively low dose could be sufficient for primary treatment [27]. Therefore, Li’s group concluded that intra-arterial administration could be a better option during gene therapy for tumors with constant blood vessel feedings, such as the cancers in head and neck regions, the liver, and the lungs, and the drugs could be delivered to the deep locations and hard tissues in order to diminish the blind spot issue of the intra-tumoral injections [27]. Moreover, some technical skills for increasing the MOI of the gene therapy drugs during different administration routes have been developed, such as removing the ascites or hydrothorax fluid before peritoneal or pleural perfusion, carrying out hepato-arterial embolization in the treatment of hepatic cancer, and using interventional treatment to localize the arterial feeding in intra-arterial perfusion.

### 2.3. Treatment Cycle of the Administration

The transfection efficiencies of the gene therapy are significantly varied by the viral vectors and the target cells, so preclinical studies of the therapy reagents are necessary, prior to the clinical trial. In our previous research of rAd-p53, we found that the expression of WT p53 and the downstream factors in tumor cells, gradually changed , reaching the highest peak 72 h after the successful transfection, before decreasing [27,59]. Due to the quick consumption of the exogenous genes, the effect of the gene therapy could not last long enough to maintain the high level of the functional genes. Therefore, we suggested repeating the transfection every 72 h after the first administration, in order to maintain the expression of p53 in the cells, ensuring an effective modification of the tumor cells. This treatment protocol was different from the one utilized in the Gendicine^TM^ study, but the results of our clinical trial showed that it was suitable for oral cancer patients, which confirmed the necessity of preclinical studies. Albeit low, the infection rate of rAd-p53 is an urgent problem that needs to be solved, in order to improve the clinical outcomes of patients with advanced cancers. Tumor-specific, replication-competent oncolytic adenoviruses, including AdDelta24-p53 [66], SG600-p53 [67], and OBP-702 [68], are being developed as novel vectors for anticancer gene therapies; in these vectors, the promoters of cancer-related genes are used to regulate virus replication in a tumor-dependent manner. The preclinical studies have shown that the replication-competent oncolytic adenoviruses are safe and effective therapies for inducing antitumor effects. Further clinical trials of the replication-competent CRAd-p53 vectors are needed for the evaluation of the effects in cancer patients [69]. Furthermore, repeated administration is essential in gene therapy. It is hard to successfully remodel malignant cells in a single transfection, and the objective will be gained over several cycles, thus the optimal treatment cycles require preclinical determination. Meanwhile, the relationship between the expression and function of target genes remain unclear. In the previous clinical trials of rAd-p53, the cycles varied from two to 10 times in one complete treatment plan, but most researchers did not clarify the rationale for these specific cycles (Table 1). The most likely reason for the cycle decision might be based on the medical direction of rAd-p53 and other treatment plans, such as radio- and chemotherapy, in the combined therapy treatment. Theoretically, the expression of the gene is the basic requirement for genetic function. Therefore, an adequate time period, wherein there is a continuously high expression of the target gene in the cancer cells, should be ensured, in order to influence the cellular genetic materials. As such, we suggest a protocol that includes at least five infection cycles to ensure effective results.

### 2.4. Evaluation of the Gene Therapy

The main purpose of traditional treatments and gene therapy are very different from each other. Traditional methods, such as surgery, and chemo- and radiotherapy, aim at diminishing the cancer by all means. The main objective of chemo-radiotherapy is to induce cellular apoptosis by ionizing DNA single- and double-strand breaks and base damage [70,71], while surgical resection aims to remove the primary or metastatic focus as completely as possible. Based on the aims of the traditional strategies, the Response Evaluation Criteria In Solid Tumors (RECIST) was published in February 2000, by an international collaboration including the European Organization for Research and Treatment of Cancer (EORTC), National Cancer Institute of the United States, and the National Cancer Institute of Canada Clinical Trials Group, and comprised a set of published rules that define when tumors in cancer patients improve (“respond”), stay the same (“stabilize”), or worsen (“progress”), during treatments. The majority of clinical trials evaluating cancer treatments for an objective response in solid tumors are using RECIST, of which the core criterion is the extent of eradicating the cancer cells and the primary focus [72].

On the other hand, the aim of gene therapy is to transform the “bad guys” back to “ordinary ones”, which means repairing the gene mutation or deletion, to end the immortality of cancer cells, and transform them back to the regular, programmed cell death. On this premise, the evaluation of gene therapy may be different from traditional methods. As more gene therapy clinical trials have been carried out, their results have not been consistent with the RECIST criteria of treatment evaluation. Following the combined treatment of gene therapy with chemo-or radiotherapy, many patients with stabilized results showed satisfying results, the cancer focuses could be detected by CT or MRI, but the subjective manifestations were diminished, the overall survival rates were significantly prolonged, and the patients were deemed to chronically live with the tumor [27]. These distinctive results might be due to the principle of gene therapy, which aims to restore the abnormal expression or dysfunction of target genes and/or the factors in the downstream signal pathway, in order to eliminate the malignant biological properties of cancer cells to remodel them back to the normal track, but not to kill the entire cells with highly proliferative abilities. So, in our clinical practice, we could not observe a rapid reduction in the volume of the primary or metastatic cancer focus in the early stages of the combined treatment, but the subjective manifestations of the patients were significantly relieved, which were different to those being observed in the traditional therapeutic strategies. Based on these findings, we suggest that the RECIST criteria are not a suitable measure for gene therapy, and a new guideline should be set out for further gene therapy clinical trials.

## 3. The Future of Gene Therapy

Gene therapy has been developed over the past 26 years, and the clinical experts have achieved a lot of experience during the clinical trials. However, there are still some problems that need to be addressed and additional areas that can be explored. First, cancer cells are known to usurp complicated networks of cell signaling; therefore, inhibition of one pathway may lead to the compensatory activation of a different pathway, which may result in treatment resistance. This could explain why the outcomes of gene therapy have not been as optimal as we have desired. So, new reagents carrying multiple genes should be developed, and should be based on the gene analysis of the target cells. Secondly, with the development of precision medicine, gene microarrays are becoming cheaper and easier to manipulate, which provides conveniences for personalized medicine. For example, the genetic mutation of p53 is a biomarker in head and neck cancer and exists in 60%–70% patients, but there are still more than 20% patients without the mutation, thus the p53 gene therapy will not be effective for this population. A pretreatment gene chip is therefore necessary for gene therapy. Furthermore, we could customize a more precise reagent of gene therapy for a single patient, based on the results of microarrays, to increase the therapeutic effect. Thirdly, by using vaccines which target tumor-associated antigens (TAA), immunotherapy may be a novel treatment strategy for cancer patients [73], which could be combined with gene therapy, such as p53 mutation in cancer cells. Although p53 mutations may represent the characteristics of TAA, most of the mutations do not occur at sites that correspond to immunological epitopes [74]. In experimental models, it has been possible to target WT p53 because the mutated molecule is associated with a high nuclear and cytoplasmic concentration of the p53 protein, and aside from point mutations, the remainder of the expressed protein is WT, which could be a good candidate for immunotherapy [75].

## 4. Conclusions

Although the development of gene therapy has not been without controversy since the beginning of the clinical trials, the results have shown that it is a promising treatment for cancer patients. Many studies are still needed to further solve theoretic and clinical issues, and to improve the therapeutic effects, but the future of gene therapy is promising and may become a basic strategy to treat patients with various diseases.

## Figures and Tables

**Table 1:** Overview of the clinical trial of rAd-p53 in China.

No.	Study ID	Time	Type of Study	Types of the Tumor	Route and Dosage of Administration	Groups	Outcome
1	Zhang S.W., et al. [22]	2001–2002	randomized controlled trial	Head and neck squamous cell carcinoma	intra-tumoral injection	Group 1 (20 patients): rAd-p53+ radiotherapy Group 2 (22 patients): radiotherapy only	CR rate: Group 1 (59%) vs. Group (22%) Fever is a common adverse effect in Group 1.
2	Zhang S.W., et al. [30]	2001–2003	randomized controlled trial	Head and neck squamous cell carcinoma	intra-tumoral injection	Group 1 (36 patients): rAd-p53+ radiotherapy Group 2 (33 patients): radiotherapy only	The CR rate of tumors in Group 2 was increased by nearly 2. 31 times more than that of tumors in Group 2. No dose limiting toxicity and adverse events were noted, except transient fever after Gendicine administration
3	Pan J.J., et al. [31]	2001–2003	randomized controlled trial	Nasopharyngeal carcinoma	intra-tumoral injection	Group 1 (42 patients): rAd-p53+ radiotherapy Group 2 (40 patients): radiotherapy only	Response rate of Group 1 was higher than that of Group 2 at each treatment point (*p* < 0.01). The 5-year overall survival rate and 5-year disease-free survival rate of Group 1 were 7.5% (*p* = 0.34) and 11.7% (*p* = 0.21) higher than those of Group 2.
4	Li Y., et al. [27]	2003–2007	randomized controlled trial	Advanced oral squamous cell carcinoma	intra-arterially infusion	Group 1 (35 patients): rAd-p53+ chemotherapy Group 2 (33 patients): rAd-p53+ placebo Group 3 (31 patients): placebo+ chemotherapy	Complete response rate: group 1 (48.5%) vs. groups 2 (16.7%) vs. group 3 (17.2%) (*p* = 0.006). Group 1 had a significantly higher survival rate than group 3 (*p* = 0.019). The survival rate of patients with stage III oral cancer was significantly higher in group 1 than in group 3 (*p* = 0.015). The most frequent vector-related complication was a transient fever.
5	Guan Y.S., et al. [25]	2004–2005	controlled clinical trial	Advanced non-small-cell lung cancer	bronchial arterial access (BAI)	Group 1 (19 patients): rAd-p53+ anti-tumor drug regimes Group 2 (39 patients): anti-tumor drug regimes	Overall response rates: Group 1 (47.3%) vs. Group 2 (38.4%) (*p* > 0.05). TTP analysis showed that Group 1 had a longer time without progression (*p* = 0.018, Log-Rank). Group 1 have less adverse events such asanorexia, nausea and emesis, pain, and leucopenia (*p* < 0.05) but more arthralgia, fever, influenza-like symptom, and myalgia (*p* < 0.05).
6	Guan Y.S., et al. [26]	2004–2005	controlled clinical trial	Advanced hepatic cell carcinoma	intra-tumoral injection	Group 1 (68 patients): rAd-p53+ TACE Group 2 (82 patients): TACE only	The total effective rate: Group 1 (58.3%) vs. Group 2 (26.5%) (*p* < 0.05). The incidence of gastrointestinal symptoms was lower in Group 1 than in Group 2 (*p* < 0.05). And Group 1 had higher survival rate (Log Rank, *p* = 0.0002)
7	Tian G., et al. [28]	2004–2007	randomized controlled trial	Advanced hepatic cell carcinoma	hepatic arterial injections	Group 1 (23 patients): rAd-p53 and 5-FU after TACE Group 2 (23 patients): TACE only	No difference in overall response rates between the two groups (*p* = 0.419). Median OS: 12.8 months in group 1 vs. 10.4 months in group 2. Time to progression: (log-rank *p* = 0.62) OS (log-rank *p* = 0.87). The most frequent vector-related complication was a transient fever (57%).
8	Yang Z.X., et al. [32]	2004–2007	Retrospective cohort study	hepatic cell carcinoma	intra-tumoral injections	Group 1 (20 patients): rAd-p53+ fSRT Group 2 (20 patients): fSRT only	The overall response rate: Group 1 (85%) vs. Group 2 (70%). The 1-year survival rates: Group 1 (90%) vs. Group 2 (70%) (*p* = 0.081). The1-year disease-free survival rates: Group 1 (85%) vs. Group 2 (65%). Fever and gastrointestinal toxicity were observed.
9	Zhu J.X., et al. [38]	2004–2008	controlled clinical trial	recurrent malignant gliomas	administered into the surgical wound	Group 1 (18 patients): rAd-p53+ surgery Group 2 (20 patients): Surgery only	Group 1 achieved significantly higher survival rate (*p* < 0.05). Fever was a common adverse event in Group 1.
10	Liu R.R., et al. [34]	2005–2006	controlled clinical trial	Recurrent nasopharyngeal carcinoma	intra-tumoral injection	Group 1 (15 patients): rAd-p53+ radiotherapy+ Chemotherapy Group 2 (15 patients): radiotherapy+ Chemotherapy	Effective rate differed in two groups: Group 1 (100%) vs. Group 2 (40%) (*p* < 0.05). Fever happened in 85.7% patients in Group 1.
11	Liu S., et al. [29]	2005–2011	randomized controlled trial	oral squamous cell carcinoma	Surgical wound surface injection	Group 1 (57 patients): rAd-p53+ radiotherapy Group 2 (50 patients): radiotherapy alone	OS did not differ in two groups (log-rank, *p* = 0.0586) but Group 1 had higher DFS (log-rank, *p* = 0.0002). Fever and flu-like symptoms were more frequently observed in Group 1.
12	Si Y.F., et al. [36]	2007–2008	randomized controlled trial	Nasopharyngeal carcinoma	intra-tumoral injection	Group 1 (14 patients): rAd-p53+ radiotherapy+ Chemotherapy Group 2 (15 patients): radiotherapy+ Chemotherapy	Group 1 (12/14) had higher CR rate than Group 2 (5/15) 3 month after treatment (*p* < 0.05). Fever is a common adverse effect in Group 1.
13	OU S.Q., et al. [35]	2007–2009	controlled clinical trial	Advanced hepatic cell carcinoma	TACE	Group 1 (60 patients): rAd-p53+ TACE Group 2 (60 patients): TACE only	In p53-positive patients, the effective rate of Group 1 (73.33%) was higher than that of Group 2 (46.67%) (*p* < 0.05). In the p53-negative patients, the effective rate in Group 1 and Group 2 was 66.67% and 60.00 %, respectively (*p* > 0.05).
14	Chen S., et al. [24]	2007–2009	randomized controlled trial	Advanced primary hepatic cell carcinoma	intra-arterially infusion	Group 1 (30 patients): rAd-p53+ hydroxycamptothecin Group 2 (18 patients): hydroxycamptothecin only	Group 1 had higher cumulative survival rate (log-rank *p* < 0.05). The most frequent vector-related complication was a transient fever. Eczema at the angles of the mouth occurred in 2 patients.
15	Cui H.M., et al. [33]	2007-2011	controlled clinical trial	Recurrent ovarian carcinoma	peritoneal perfusion	Group 1 (25 patients): rAd-p53+ chemotherapy Group 2 (24 patients): chemotherapy only	Disease control rate: Group 1 (92.0%)vs. Group 2 (87.5%) (*p* = 0.59), No statistical significance in overall survival between the two groups (log rank, *p* = 0.051). No serious adverse events were observed in either of the two groups.
16	Wang J.G., et al. [37]	2010–2011	randomized controlled trial	advanced non-small-cell lung cancer	bronchial arterial access	Group 1 (31 patients): rAd-p53+ radiotherapy+ Chemotherapy Group 2 (33 patients): radiotherapy+ Chemotherapy	Effective rates: Groups 1 (70.97%) vs. Group 2 (45.45%) (*p* < 0.05). Survival rates in one year: Group 1 (74.19%) vs. Group 2 (69.70%) (*p* > 0.05). Fever was a common adverse event in Group 1.

## References

[1] Wirth T., Parker N., Yla-Herttuala S. (2013). History of gene therapy. Gene.

[2] Tatum E.L. (1966). Molecular biology, nucleic acids, and the future of medicine. Perspect. Biol. Med..

[3] Rogers S., Pfuderer P. (1968). Use of viruses as carriers of added genetic information. Nature.

[4] Friedmann T., Roblin R. (1972). Gene therapy for human genetic disease?. Science.

[5] Blaese R.M., Culver K.W., Miller A.D., Carter C.S., Fleisher T., Clerici M., Shearer G., Chang L., Chiang Y., Tolstoshev P. (1995). T lymphocyte-directed gene therapy for ADA-SCID: Initial trial results after 4 years. Science.

[6] Bordignon C., Notarangelo L.D., Nobili N., Ferrari G., Casorati G., Panina P., Mazzolari E., Maggioni D., Rossi C., Servida P. (1995). Gene therapy in peripheral blood lymphocytes and bone marrow for ADA-immunodeficient patients. Science.

[7] Mukhopadhyay T., Roth J.A. (1993). A codon 248 p53 mutation retains tumor suppressor function as shown by enhancement of tumor growth by antisense p53. Cancer Res..

[8] Zhang W.W., Alemany R., Wang J., Koch P.E., Ordonez N.G., Roth J.A. (1995). Safety evaluation of Ad5CMV-p53 in vitro and in vivo. Hum. Gene Ther..

[9] Roth J.A., Fossella F., Komaki R., Ryan M.B., Putnam J.B., Lee J.S., Dhingra H., de Caro L., Chasen M., McGavran M. (1994). A randomized trial comparing perioperative chemotherapy and surgery with surgery alone in resectable stage IIIA non-small-cell lung cancer. J. Natl. Cancer Inst..

[10] Cavazzana-Calvo M., Hacein-Bey S., de Saint Basile G., Gross F., Yvon E., Nusbaum P., Selz F., Hue C., Certain S., Casanova J.L. (2000). Gene therapy of human severe combined immunodeficiency (SCID)-X1 disease. Science.

[11] Stolberg S.G. (1999). The biotech death of Jesse Gelsinger. N. Y. Times Mag..

[12] Yla-Herttuala S. (2012). Endgame: Glybera finally recommended for approval as the first gene therapy drug in the European Union. Mol. Ther..

[13] Touzot F., Moshous D., Creidy R., Neven B., Frange P., Cros G., Caccavelli L., Blondeau J., Magnani A., Luby J.M. (2015). Faster T-cell development following gene therapy compared with haploidentical HSCT in the treatment of SCID-X1. Blood.

[14] Booth C., Gaspar H.B., Thrasher A.J. (2016). Treating Immunodeficiency through HSC Gene Therapy. Trends Mol. Med..

[15] Fukuhara H., Ino Y., Todo T. (2016). Oncolytic virus therapy: A new era of cancer treatment at dawn. Cancer Sci..

[16] Almasbak H., Aarvak T., Vemuri M.C. (2016). CAR T Cell Therapy: A Game Changer in Cancer Treatment. J. Immunol. Res..

[17] Gill S., June C.H. (2015). Going viral: Chimeric antigen receptor T-cell therapy for hematological malignancies. Immunol. Rev..

[18] Louis C.U., Savoldo B., Dotti G., Pule M., Yvon E., Myers G.D., Rossig C., Russell H.V., Diouf O., Liu E. (2011). Antitumor activity and long-term fate of chimeric antigen receptor-positive T cells in patients with neuroblastoma. Blood.

[19] Ahmed N., Brawley V.S., Hegde M., Robertson C., Ghazi A., Gerken C., Liu E., Dakhova O., Ashoori A., Corder A. (2015). Human Epidermal Growth Factor Receptor 2 (HER2)-Specific Chimeric Antigen Receptor-Modified T Cells for the Immunotherapy of HER2-Positive Sarcoma. J. Clin. Oncol..

[20] Feng K., Guo Y., Dai H., Wang Y., Li X., Jia H., Han W. (2016). Chimeric antigen receptor-modified T cells for the immunotherapy of patients with EGFR-expressing advanced relapsed/refractory non-small cell lung cancer. Sci. China Life Sci..

[21] Beatty G.L., Haas A.R., Maus M.V., Torigian D.A., Soulen M.C., Plesa G., Chew A., Zhao Y., Levine B.L., Albelda S.M. (2014). Mesothelin-specific chimeric antigen receptor mRNA-engineered T cells induce anti-tumor activity in solid malignancies. Cancer Immunol. Res..

[22] Zhang S.W., Xiao S.W., Liu C.Q., Sun Y., Su X., Li D.M., Xu G., Cai Y., Zhu G.Y., Xu B. (2003). Treatment of head and neck squamous cell carcinoma by recombinant adenovirus-p53 combined with radiotherapy: A phase II clinical trial of 42 cases. Zhonghua Yi Xue Za Zhi.

[23] Peng Z. (2005). Current status of gendicine in China: Recombinant human Ad-p53 agent for treatment of cancers. Hum. Gene Ther..

[24] Chen S., Chen J., Xi W., Xu W., Yin G. (2014). Clinical therapeutic effect and biological monitoring of p53 gene in advanced hepatocellular carcinoma. Am. J. Clin. Oncol..

[25] Guan Y.S., Liu Y., Zou Q., He Q., La Z., Yang L., Hu Y. (2009). Adenovirus-mediated wild-type p53 gene transfer in combination with bronchial arterial infusion for treatment of advanced non-small-cell lung cancer, one year follow-up. J. Zhejiang Univ. Sci. B.

[26] Guan Y.S., Liu Y., He Q., Li X., Yang L., Hu Y., La Z. (2011). p53 gene therapy in combination with transcatheter arterial chemoembolization for HCC: One-year follow-up. World J. Gastroenterol..

[27] Li Y., Li L.J., Wang L.J., Zhang Z., Gao N., Liang C.Y., Huang Y.D., Han B. (2014). Selective intra-arterial infusion of rAd-p53 with chemotherapy for advanced oral cancer: A randomized clinical trial. BMC Med..

[28] Tian G., Liu J., Zhou J.S., Chen W. (2009). Multiple hepatic arterial injections of recombinant adenovirus p53 and 5-fluorouracil after transcatheter arterial chemoembolization for unresectable hepatocellular carcinoma: A pilot phase II trial. Anticancer Drugs.

[29] Liu S., Chen P., Hu M., Tao Y., Chen L., Liu H., Wang J., Luo J., Gao G. (2013). Randomized, controlled phase II study of post-surgery radiotherapy combined with recombinant adenoviral human p53 gene therapy in treatment of oral cancer. Cancer Gene Ther..

[30] Zhang S.W., Xiao S.W., Liu C.Q., Sun Y., Su X., Li D.M., Xu G., Zhu G.Y., Xu B. (2005). Recombinant adenovirus-p53 gene therapy combined with radiotherapy for head and neck squamous-cell carcinoma. Zhonghua Zhong Liu Za Zhi.

[31] Pan J.J., Zhang S.W., Chen C.B., Xiao S.W., Sun Y., Liu C.Q., Su X., Li D.M., Xu G., Xu B. (2009). Effect of recombinant adenovirus-p53 combined with radiotherapy on long-term prognosis of advanced nasopharyngeal carcinoma. J. Clin. Oncol..

[32] Yang Z.X., Wang D., Wang G., Zhang Q.H., Liu J.M., Peng P., Liu X.H. (2010). Clinical study of recombinant adenovirus-p53 combined with fractionated stereotactic radiotherapy for hepatocellular carcinoma. J. Cancer Res. Clin. Oncol..

[33] Cui H.M., Guan C.L., Liu Q., Li L.Y. (2014). Outcome of patients with recurrent epithelial ovarian carcinoma following treatment with recombinant human adenovirus p53 combined with chemotherapy. Chin. J. Cancer Biother..

[34] Liu R.R., Ji C.Y., Chen J.C. (2010). Clinical effect of recombinant human p53 adv injection (gendicine) in combination with radiotherapy in patients suffering from recurrent nasopharyngeal carcinoma. J. Otolarngol. Ophthal. Shandong Univ..

[35] Ou S.Q., Ma Y.L., Kang P., Li Z.K., Meng Z.B.F.Q. (2010). Recombinant adenovirus-p53 gene therapy combined with transcatheter arterial chemoembolization for p53-positive and p53-negative hepatocellular carcinoma. Chin. J. Interv. Imaging Ther..

[36] Si Y.F., He C.C., Lan G.P., Huang B., Zhang Z., Lu J.L., Zhou R.J., Jiang H. (2009). Recombinant Adenovirus P53 Agent Injection Combined with Radiotherapy and Chemotherapy for Intermediate and Advanced Stage Nasopharyngeal Carcinoma. ZhongGuo Zhong Liu Lin Chuang.

[37] Wang J.G., Wang X.H., Yang J.Q., Li G.H., Hu W.N. (2014). Treatment of Local Advanced Non-small Cell Lung Cancer with Recombinant Human p53 Adenovirus Combined with Radiochemotherapy. J. GuiYang Med. Coll..

[38] Zhu J.X., Li Z.M., Geng F.Y., Fu Q., Guo C.J., Xiao Y.L., Zhang Z.T., Li G. (2010). Treatment of recurrent malignant gliomas by surgery combined with recombinant adenovirus-p53. Chin. J. Cancer Prev. Treat..

[39] Yoo G.H., Moon J., Leblanc M., Lonardo F., Urba S., Kim H., Hanna E., Tsue T., Valentino J., Ensley J. (2009). A phase 2 trial of surgery with perioperative INGN 201 (Ad5CMV-p53) gene therapy followed by chemoradiotherapy for advanced, resectable squamous cell carcinoma of the oral cavity, oropharynx, hypopharynx, and larynx: Report of the Southwest Oncology Group. Arch. Otolaryngol. Head Neck Surg..

[40] Gabrilovich D.I. (2006). INGN 201 (Advexin): Adenoviral p53 gene therapy for cancer. Expert Opin. Biol. Ther..

[41] Wolf J.K., Bodurka D.C., Gano J.B., Deavers M., Ramondetta L., Ramirez P.T., Levenback C., Gershenson D.M. (2004). A phase I study of Adp53 (INGN 201; ADVEXIN) for patients with platinum- and paclitaxel-resistant epithelial ovarian cancer. Gynecol. Oncol..

[42] Saulnier P., Vidaud M., Gautier E., Motte N., Bellet D., Escudier B., Wilson D., Yver A. (2003). Development and validation of a real-time PCR assay for the detection and quantitation of p53 recombinant adenovirus in clinical samples from patients treated with Ad5CMV-p53 (INGN 201). J. Virol. Methods.

[43] Swisher S.G., Roth J.A., Komaki R., Gu J., Lee J.J., Hicks M., Ro J.Y., Hong W.K., Merritt J.A., Ahrar K. (2003). Induction of p53-regulated genes and tumor regression in lung cancer patients after intratumoral delivery of adenoviral p53 (INGN 201) and radiation therapy. Clin. Cancer Res..

[44] Merritt J.A., Roth J.A., Logothetis C.J. (2001). Clinical evaluation of adenoviral-mediated p53 gene transfer: Review of INGN 201 studies. Semin. Oncol..

[45] Nemunaitis J., Clayman G., Agarwala S.S., Hrushesky W., Wells J.R., Moore C., Hamm J., Yoo G., Baselga J., Murphy B.A. (2009). Biomarkers Predict p53 Gene Therapy Efficacy in Recurrent Squamous Cell Carcinoma of the Head and Neck. Clin. Cancer Res..

[46] Zeng Q., Wang J., Lv X., Li J., Yin L.J., Xiang Y.Q., Guo X. (2016). Induction Chemotherapy Followed by Radiotherapy versus Concurrent Chemoradiotherapy in elderly patients with nasopharyngeal carcinoma: Finding from a propensity-matched analysis. BMC Cancer.

[47] Mitsudo K., Koizumi T., Iida M., Iwai T., Nakashima H., Oguri S., Kioi M., Hirota M., Koike I., Hata M. (2014). Retrograde superselective intra-arterial chemotherapy and daily concurrent radiotherapy for stage III and IV oral cancer: Analysis of therapeutic results in 112 cases. Radiother. Oncol..

[48] Strigari L., Mancuso M., Ubertini V., Soriani A., Giardullo P., Benassi M., D’Alessio D., Leonardi S., Soddu S., Bossi G. (2014). Abscopal effect of radiation therapy: Interplay between radiation dose and p53 status. Int. J. Radiat. Biol..

[49] Lu C., El-Deiry W.S. (2009). Targeting p53 for enhanced radio- and chemo-sensitivity. Apoptosis.

[50] Kranz D., Dobbelstein M. (2012). A killer promoting survival: p53 as a selective means to avoid side effects of chemotherapy. Cell Cycle.

[51] El-Deiry W.S. (2003). The role of p53 in chemosensitivity and radiosensitivity. Oncogene.

[52] Farrand L., Kim J.Y., Byun S., Im-aram A., Lee J., Suh J.Y., Lee K.W., Lee H.J., Tsang B.K. (2014). The diarylheptanoid hirsutenone sensitizes chemoresistant ovarian cancer cells to cisplatin via modulation of apoptosis-inducing factor and X-linked inhibitor of apoptosis. J. Biol. Chem..

[53] Hamada M., Fujiwara T., Hizuta A., Gochi A., Naomoto Y., Takakura N., Takahashi K., Roth J.A., Tanaka N., Orita K. (1996). The p53 gene is a potent determinant of chemosensitivity and radiosensitivity in gastric and colorectal cancers. J. Cancer Res. Clin. Oncol..

[54] Cheung K.J., Horsman D.E., Gascoyne R.D. (2009). The significance of TP53 in lymphoid malignancies: Mutation prevalence, regulation, prognostic impact and potential as a therapeutic target. Br. J. Haematol..

[55] Berge E.O., Huun J., Lillehaug J.R., Lonning P.E., Knappskog S. (2013). Functional characterisation of p53 mutants identified in breast cancers with suboptimal responses to anthracyclines or mitomycin. Biochim. Biophys. Acta.

[56] Sakai K., Kazama S., Nagai Y., Murono K., Tanaka T., Ishihara S., Sunami E., Tomida S., Nishio K., Watanabe T. (2014). Chemoradiation provides a physiological selective pressure that increases the expansion of aberrant TP53 tumor variants in residual rectal cancerous regions. Oncotarget.

[57] Jiang G., Xin Y., Zheng J.N., Liu Y.Q. (2011). Combining conditionally replicating adenovirus-mediated gene therapy with chemotherapy: A novel antitumor approach. Int. J. Cancer.

[58] Prados J., Alvarez P.J., Melguizo C., Rodriguez-Serrano F., Carrillo E., Boulaiz H., Velez C., Marchal J.A., Caba O., Ortiz R. (2012). How is gene transfection able to improve current chemotherapy? The role of combined therapy in cancer treatment. Curr. Med. Chem..

[59] Li Y., Li B., Li C.J., Li L.J. (2015). Key points of basic theories and clinical practice in rAd-p53 (Gendicine) gene therapy for solid malignant tumors. Expert Opin. Biol. Ther..

[60] Lowenstein P.R. (2003). Virology and immunology of gene therapy, or virology and immunology of high MOI infection with defective viruses. Gene Ther..

[61] Alemany R. (2007). Cancer selective adenoviruses. Mol. Aspects Med..

[62] Li Y., Li L.J., Zhang S.T., Wang L.J., Zhang Z., Gao N., Zhang Y.Y., Chen Q.M. (2009). In vitro and clinical studies of gene therapy with recombinant human adenovirus-p53 injection for oral leukoplakia. Clin. Cancer Res..

[63] Moon C., Oh Y., Roth J.A. (2003). Current status of gene therapy for lung cancer and head and neck cancer. Clin. Cancer Res..

[64] Van Zeeburg H.J., Huizenga A., Brink A., van den Doel P.B., Zhu Z.B., McCormick F., Brakenhoff R.H., van Beusechem V.W. (2010). Comparison of oncolytic adenoviruses for selective eradication of oral cancer and pre-cancerous lesions. Gene Ther..

[65] Clayman G.L., Frank D.K., Bruso P.A., Goepfert H. (1999). Adenovirus-mediated wild-type p53 gene transfer as a surgical adjuvant in advanced head and neck cancers. Clin. Cancer Res..

[66] Van Beusechem V.W., van den Doel P.B., Grill J., Pinedo H.M., Gerritsen W.R. (2002). Conditionally replicative adenovirus expressing p53 exhibits enhanced oncolytic potency. Cancer Res..

[67] Wang X., Su C., Cao H., Li K., Chen J., Jiang L., Zhang Q., Wu X., Jia X., Liu Y. (2008). A novel triple-regulated oncolytic adenovirus carrying p53 gene exerts potent antitumor efficacy on common human solid cancers. Mol. Cancer Ther..

[68] Yamasaki Y., Tazawa H., Hashimoto Y., Kojima T., Kuroda S., Yano S., Yoshida R., Uno F., Mizuguchi H., Ohtsuru A. (2012). A novel apoptotic mechanism of genetically engineered adenovirus-mediated tumour-specific p53 overexpression through E1A-dependent p21 and MDM2 suppression. Eur. J. Cancer.

[69] Tazawa H., Kagawa S., Fujiwara T. (2013). Advances in adenovirus-mediated p53 cancer gene therapy. Expert Opin. Biol. Ther..

[70] Koom W.S., Park S.Y., Kim W., Kim M., Kim J.S., Kim H., Choi I.K., Yun C.O., Seong J. (2012). Combination of radiotherapy and adenovirus-mediated p53 gene therapy for MDM2-overexpressing hepatocellular carcinoma. J. Radiat. Res..

[71] Morris J.C. (2003). Cancer gene therapy: Lessons learned from experiences with chemotherapy. Mol. Ther..

[72] Schwartz L.H., Litiere S., de Vries E., Ford R., Gwyther S., Mandrekar S., Shankar L., Bogaerts J., Chen A., Dancey J. (2016). RECIST 1.1-Update and clarification: From the RECIST committee. Eur. J. Cancer.

[73] Feng D., Shaikh A.S., Wang F. (2016). Recent Advance in Tumor-associated Carbohydrate Antigens (TACAs)-based Antitumor Vaccines. ACS Chem. Biol..

[74] Wiedenfeld E.A., Fernandez-Vina M., Berzofsky J.A., Carbone D.P. (1994). Evidence for selection against human lung cancers bearing p53 missense mutations which occur within the HLA A*0201 peptide consensus motif. Cancer Res..

[75] Zhou S.L., Yue W.B., Fan Z.M., Du F., Liu B.C., Li B., Han X.N., Ku J.W., Zhao X.K., Zhang P. (2014). Autoantibody detection to tumor-associated antigens of P53, IMP1, P16, cyclin B1, P62, C-myc, Survivn, and Koc for the screening of high-risk subjects and early detection of esophageal squamous cell carcinoma. Dis. Esophagus.

